# β-amyloid wall deposit of temporal artery in subjects with spontaneous intracerebral haemorrhage

**DOI:** 10.18632/oncotarget.26165

**Published:** 2018-10-05

**Authors:** Antonino Tuttolomondo, Rosario Maugeri, Elisabetta Orlando, Giulio Giannone, Francesco Ciccia, Aroldo Rizzo, Domenico Di Raimondo, Francesca Graziano, Rosaria Pecoraro, Carlo Maida, Irene Simonetta, Anna Cirrincione, Francesca Portelli, Francesca Corpora, Domenico Gerardo Iacopino, Antonio Pinto

**Affiliations:** ^1^ Internal Medicine and Stroke Care Ward, Dipartimento Biomedico di Medicina Interna e Specialistica, University of Palermo, Palermo, Italy; ^2^ Human Pathology Section, Human Pathology Section, Department of Health Sciences, University of Palermo, Palermo, Italy; ^3^ Rheumathology Ward, Dipartimento Biomedico di Medicina Interna e Specialistica, University of Palermo, Palermo, Italy; ^4^ Neurosurgery Ward, Dipartimento di BioMedicina Sperimentale e Neuroscienze Cliniche, Università degli Studi di Palermo, Palermo, Italy; ^5^ Human Pathology Section, Azienda Ospedaliera Ospedali Riuniti Villa Sofia-Cervello, Palermo, Italy

**Keywords:** β-amyloid, superficial temporal artery, intracerebral haemorrhage, CAAH

## Abstract

**Background:**

Cerebral Amyloid Angiopathy has been indicated as an important cause of spontaneous non-hypertensive intracerebral haemorrhage (ICH).

**Aims:**

to analyze the presence of β-amyloid deposit in the temporal artery of consecutive patients with ICH in comparison to control subjects and its relation to APO-E haplotype frequency.

**Methods:**

We enrolled consecutive patients admitted to Neurosurgery Ward of University Hospital “P. Giaccone” of Palermo with a diagnosis of spontaneous non hypertensive ICH and as control 12 subjects without brain haemorrhage. Biopsy of superficial temporal artery has been performed and β-amyloid deposit was quantified.

**Results:**

Among 25 subjects with ICH, 10 (40%) had APOE epsilon 2 allele and among these subjects 7 (70%) showed amyloid accumulation on temporal artery specimens, 8 (32%) subjects had APOE epsilon 3 allele and among these subjects only 2 (25%) showed amyloid accumulation on temporal artery specimens, whereas 7 (28%) had APOE epsilon 4 allele and of these, 7 (100%) showed amyloid accumulation on temporal artery specimens. At multivariable logistic regression analysis for the presence of amyloid, predictive factors for the presence of amyloid in temporal artery biopsies were: age, hypertension, intralobar site of haemorrhage, APOE epsilon 2 and APOE epsilon 4 alleles.

**Discussion:**

Our findings of a higher frequency of amyloid deposition in temporal artery specimens in subjects with spontaneous intracerebral haemorrhage indicate a possible role of temporal artery as a possible diagnostic site of biopsy in subjects at high risk to develop intracranial haemorrhage related to Cerebral Amyloid Angiopathy.

## INTRODUCTION

Spontaneous non-traumatic intracerebral haemorrhage (ICH) is the second most prevalent subtype of stroke, and it has been associated with high mortality and morbidity throughout the world [1, 2]. It usually results from the rupture of small arteries in the brain and represents 10–30% of all strokes [3]. In recent years, Cerebral Amyloid Angiopathy (CAA) has been rediscovered as a common cause of spontaneous ICH [4, 5]. CAA refers to the deposition of β-amyloid (Aβ) in the media and adventitia of small and mid-sized arteries of the cerebral cortex and the leptomeninges [4].

Some authors described the pathological features associated with CAA: -preferential involvement of the small arteries and capillaries of the meninges, cerebral cortex, and cerebellar cortex [6]; -a topographical distribution favouring the posterior brain regions, most frequently involving the occipital lobes [7]; -lack of staining of vessels in the white matter [8]; -association with increased age and the presence of dementia [9]; -lack of association with hypertension and arteriosclerosis [10]; -lack of association with amyloidosis of the other organs [11, 12].

Pathologically defined CAA is common in the elderly. Population based autopsy studies indicate a CAA prevalence of 20–40% in non-demented and 50–60% in demented elderly populations [13–17]. Advancing age is the strongest known clinical risk factor for developing CAA [13–15, 18–22, 25].

In contrast with hypertensive arteriopathy, the other form of small vessel disease and cause of ICH, the risk of CAA is not accounted for by conventional cardiovascular risk factors other than age [23]. Hypertension is not considered a risk factor for developing CAA but it may increase the risk of CAA related ICH [23].

The temporal artery is a blood vessel in the scalp on the side of the head and superficial temporal artery is a major artery of the head. It arises from the external carotid artery when it bifurcates into the superficial temporal artery and maxillary artery. Temporal artery biopsy (TAB) is a procedure that involves removing a small section of the temporal artery and it has previously been considered fundamental to making the diagnosis in clinical setting of giant cell arteritis (GCA). Few studies analyzed the potential role of TAB in other diseses such as those with pathological accumulation on brain vessels such as CAA. A single study reported a case of amyloidosis AL associated with light lambda chain myeloma, mimicking giant cell temporal arteritis [24].

No study has evaluated the presence of β-amyloid deposits in the temporal artery in subjects with intracerebral haemorrhage. The demonstration of β-amyloid accumulation in bioptic specimens from temporal artery may represent a useful clinical opportunity since the easier surgical access also *in vivo* to this arterial site.

Our study hypothesis was that in subjects with non-hypertensive brain haemorrhage the histological analysis of specimens obtained by TAB could offer useful diagnostic information towards a possible *ex-vivo* diagnosis of CAA based on the presence of β-amyloid accumulation on the wall of superficial temporal artery.

Thus, we designed a case control study with these aims: -to evaluate the presence of β-amyloid deposit in temporal artery specimens of consecutive patients with spontaneous non-hypertensive intracerebral haemorrhage in comparison to control subjects; -to assess the frequency of the APO-E alleles in subjects with spontaneous non-hypertensive intracerebral haemorrhage with regard to β-amyloid accumulation in temporal artery specimens; -to analyse the predictive role of clinical, laboratory and genetic variables towards the presence of β-amyloid accumulation in temporal artery biopsies.

## RESULTS

We enrolled 25 consecutive subjects with spontaneous intracerebral haemorrhage.

General and clinical characteristics of patients with spontaneous intracerebral haemorrhage were listed in Table 1. Mean age was 66 ± 5.19 years. 16 were males and 9 were females,14 (56%) had hypertension, 5 (20%) had diabetes, 8 (32%) had dyslipidemia, 4 (16%) had a previous stroke, 5 (20%) had a previous smoking habit.

**Table 1:** Baseline characteristics in patients with intracerebral spontaneous haemorrhage and in control subjects

	Patients with intracerebral haemorrhages (*n*: 25)	Control subjects (*n*: 12)
Age	66 ± 5.19	67 ± 6.06
Sex (M/F) (*n*%)	16/9 (64/36)	7/5 (58.3/42.7)
Hypertension (*n*%)	14 (56)	7 (58.33)
Diabete (*n*%)	5 (20)	4 (33.3)
Dyslipidemia	8 (32)	4 (33.3)
Previous stroke	4 (16)	2 (16.6)
Smoking current	5 (20)	2 (16.6)
Smoking previous	4 (16)	2 (16.6)
Serum glucose (mg/dL)	128.1 ± 23.1	121.1 ± 14.3
Total cholesterol (mg/dL)	189.5 ± 37.4	179.5 ± 41.4
HDL cholesterol (mg/dL)	57.0 ± 18.6	49.0 ± 18.6
LDL cholesterol (mg/dL)	101.7 ± 13.4	109.5 ± 21.7
Triglyceride (mg/dL)	122.8 ± 51.5	131.4 ± 37.1
Serum creatinine (mg/dL)	0.95 ± 0.18	0.89 ± 0.31
eGFR (mL/min/1.73 m2)	88.4 ± 22.5	89.8 ± 13.2
Location of ICH (*n*%)		−
- Ganglionic	4 (16)	−
- Lobar	18 (72)	−
- brainstem/cerebellum	3 (12)	
Amyloid accumulation	19 (76)	3 (25)
CAA severity according the		
Vonsattel scale	**2.6** ± **0.9**	**1.1** ± **0.8**
(mean ± ds)		
CAA severity according the Mountjoy scale (mean ± ds)	**3.56** ± **1.1**	**1.38** ± **0.7**
Apo-E alleles (*n*%)		
*-epsilon 2 allele*	**10 (40%)**	−
*-epsilon 3 allele*	**8 (32%)**	**3 (25)**
*-epsilon 4 allele*	**7 (28%)**	−

With regard to ICH localization, 18 (72%) had a lobar intracerebral haemorrhage and 7 (28%) had a non-lobar intracerebral haemorrhage (4 ganglionic and 3 brainstem/cerebellum).

We observed a β-amyloid deposit in 19 subjects with ICH and in 3 control subjects (see Table 1).

**Figure 1:**
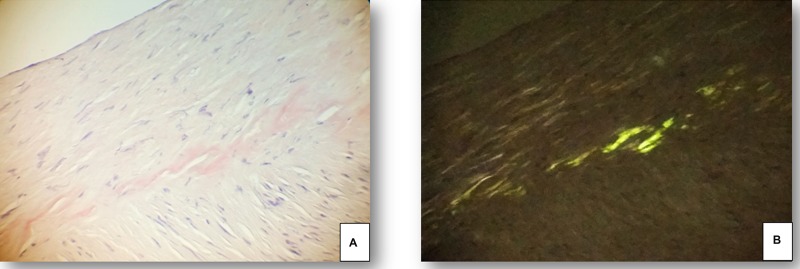
Superficial temporal artery specimen from a 67 year old male patient with spontaneous intracerebral haemorrhage and apo ε_2_ genotype (**A**) Congophilic amorphous material in the muscular wall of the superficial temporal artery. (**B**) The polarized light evidences a linear “apple green” birefringence, related to amyloid deposites. (Congo Red, 100x).

**Figure 2:**
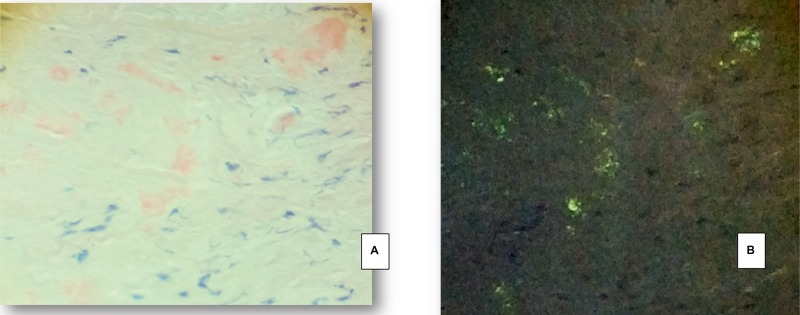
Superficial temporal artery specimen from a 68 year old female patient with spontaneous intracerebral haemorrhage and apo ε_4_ genotype (**A**) Congophilic amorphous material in the muscular wall of the superficial temporal artery. (**B**) The polarized light evidences week granular “apple green” birefringence, related to amyloid deposites. (A, B: Congo Red, stain 200x).

Subjects with ICH in comparison with controls showed a higher severity of amyloid accumulation either according the Vonsattel scale (2.6 ± 0.9 vs 1.1 ± 0.8) and a higher percentage of β-amyloid deposit measured by Mountjoy scale (3.56 ± 1.1 vs 1.38 ± 0.7) (see Tables 1 and 2 and Figures 1–2).

**Table 2:** Cerebral amyloid angiopathy severity in temporal arterie of patients with intracerebral haemorrhages

Number/initials	age	sex	CAA severity according the Vonsattel scale	CAA severity according the Mountjoi scale
1	67	M	1	3
2	71	M	3	4
	66	F	3	4
4	68	M	3	4
5	59	F	3	4
7	68	F	3	4
8	70		3	4
9	65	M	3	4
10	68	M	3	4
11	73	F	3	4
12	57	F	3	3
13	61	F	3	3
14	59	M	2	4
15	66	M	3	3
16	65	M	3	4
17	63	F	3	4
18	62	F	3	4
19	59	F	2	4
20	57	M	3	4
21	77	M	3	4
22	81	M	1	3
23	64	F	3	3
24	62	M	3	3
25	69	M	3	4

Apolipoprotein E allele frequencies in subjects with ICH were: 10 (40%) had APOE epsilon 2 allele and among these subjects 7 (70%) showed β-amyloid accumulation on temporal artery specimens, 8 (32%) had APOE epsilon 3 allele and among these subjects only 2 (25%) showed β-amyloid accumulation on temporal artery specimens, 7 (28%) had APOE epsilon 4 allele and of these 100 (%) showed β-amyloid accumulation on temporal artery specimens.

Among subjects without spontaneous intracerebral haemorrhage only 3 subjects showed β-amyloid accumulation on temporal artery specimens and all these subjects had APOE epsilon 3 allele (see Table 1).

At multivariable logistic regression analysis for presence of β-amyloid in temporal artery specimens of patients with spontaneous ICH, predictive variables for the presence of β-amyloid in temporal artery biopsies were: age (per 1 year) (OR: 1.48; *p* = 0.035), hypertension (OR: 2.23; *p* = 0.085), intralobar site of haemorrhage (OR: 1.66; *p* = 0.03), APOE epsilon 2 allele (OR: 1.96; *p* < 0.0001**),** APOE epsilon 4 allele (OR: 1.88; *p* < 0.0001) (see Table 3).

**Table 3:** Multivariable logistic regression analysis for presence of amyloid in temporal artery specimens of patients with spontaneous intracerebral haemorrhage

	OR	95% CI	*p*-value
Age (per 1 year)	1.48	1.01–1.99	**0.035**
Hypertension	2.23	0.83–5.05	**0.085**
diabetes	2.09	1.12–8.56	**0.03**
Smoking	1.03	0.51–5.42	0.408
eGFR	0.99	0.98–1.01	0.689
Intralobar	1.66	0.61–4.56	**0.03**
Prior stroke	0.67	0.61–2.34	0.120
Dyslipidemia	0.74	0.55–1.76	0.609
Total cholesterol	0.56	0.48–2.11	0.709
Triglyceride	0.88	0.58–1.97	0.505
APOE epsilon 2 allele	1.96	1.11–2.52	**< 0.0001**
APOE epsilon 4 allele	1.88	1.10–2.35	**< 0.0001**

## DISCUSSION

We report that in subjects with spontaneous intracerebral haemorrhage, histological analysis of temporal artery specimens show an accumulation of β-amyloid. In subjects with intracerebral haemorrhage in comparison with controls we also observed a higher severity of amyloid accumulation either according the Vonsattel and the Mountjoy scales.

To our best knowledge, there are no reports concerning the temporal artery involvement in CAA or ICH. Therefore, we tried to find out how often the temporal artery are deposited by β-amyloid in subjects with ICH due to non-hypertensive spontaneous intracerebral haemorrhage. We report that this arterial site is more likely to be interested by β-amyloid deposit in subjects with ICH in comparison with control subjects.

Furthermore, concerning APO-E aplotype we tried to speculate if β-amyloid accumulation could be related to the presence of CAA.

Neuropathological examination is still a “gold standard” for CAA diagnosis but no study, to the best of our knowledge, examined the role of temporal artery biopsy in the diagnosis of β-amyloid accumulation and its practical application to perform diagnosis of CAA in subjects with organ complications such as cerebral amyloid angiopathy haemorrhage (CAAH).

Cerebral amyloid angiopathy is characterised by deposition of amyloid β-protein (Aβ) within the walls of medium and small-sized arteries in the cerebral cortex and leptomeninges [1]. These pathological changes lead to increased vascular fragility and predisposes to rupture of the blood vessel.

Our finding of a higher rate of amyloid accumulation in temporal artery specimens could offer a new diagnostic tool for CAA and a possible method to stratify population at risk of CAAH. Our results seem to sustain the role of a possible diagnosis of CAA based on histological analysis of temporal artery specimen. Thus, it could be possible to hypothesize an underestimation of CAA owing to the low percentage of patients that underwent post-mortem to pathological and histological screening for CAA and that an extended use of TAB could show an increased CAA prevalence.

Vinters [25] in a clinico-pathological series of 107 pathologically proven CAA cases found the prevalence of hypertension to be around 32%, similar to community-dwelling elderly populations, while another pathological study [26] reported that CAA patients with ICH were more frequently hypertensive (50%) than those without ICH (23%), suggesting that hypertension may contribute to CAA related cerebral bleeding.

In a recent multicentre cohort of patients with spontaneous ICH, some authors found that the prevalence of hypertension in CAA related ICH was 62% significantly less than in non-CAA related ICH (85%) [27–30].

Individuals carrying the Apo-epsilon 2 allele also have an increased risk of CAA related lobar ICH [38]. Both of these risk alleles have been also reported as associated with a younger age of first ICH, [39] greater likelihood of hematoma expansion, poorer clinical outcome [30–38] and a higher risk of recurrence [39]. Patients with both Apo epsilon 2 and 4 alleles have the earliest disease onset and high risk of early ICH recurrence [40–42].

The evidence available to date concerning a possible association between apoE genotype and haemorrhage due to CAA (CAAH) are not univocal. Some studies reported a high APOE epsilon 4 allele frequency in such patients [43–45] whereas others a high frequency of APOE epsilon 2 allele [46, 47]. More than 40% of patients with CAA-related haemorrhage have associated AD [48, 49], which may confound the analysis because APOE epsilon 4 allele is a well-established risk factor for AD [50–53], whereas APOE epsilon 2 allele is protective [54, 55].

It has been reported by some authors that CAA-associated vasculopathic complications precede vessel rupture and cerebral haemorrhage [56–58].

Two studies have reported that the APOE epsilon 2 allele allele is associated with some of these vascular complications. Among 75 brains with CAA, Greenberg *et al*. [59, 60] found an elevated APOE epsilon 2 allele frequency in brains that demonstrated both vessel wall cracking and evidence of paravascular blood leak compared to brains without this combination of vasculopathies.

Another study recently analyzed CAA-associated vasculopathic complications among CAA patients with and without macroscopic evidence of lobar haemorrhage [61]. The authors reported that stenosed blood vessels, dilated/microaneurysmal vessels and fibrinoid necrosis were more common in patients with CAAH than in patients with CAA but without macroscopic lobar haemorrhage. Thus, authors suggested that this structural change in the vessel wall may represent the pathogenetic link between APOE epsilon 2 allele and CAAH [61–63].

Our findings of a higher frequency of β-amyloid deposition in temporal artery specimens in subjecs with spontaneous intracerebral haemorrhage indicate a possible role of temporal artery as a marker arterial site of CAA in subjects at high risk to develop CAAH.

Furthermore our finding of a higher prevalence of APOE epsilon 2 allele and APOE epsilon 4 allele in subjects with spontaneous intracerebral haemorrhage and β-amyloid accumulation on temporal artery biopsy specimen could suggest that temporal artery should be considered as a possible arterial site of amyloid accumulation thus to represent a candidate diagnostic marker of CAA in subjects with predisposing APOE genotypes. Furthermore, future studies addressing analysis β-amyloid accumulation on temporal artery specimens could also offer interesting perspectives about the risk of rebleeding in subjects with ICH.

### Limitations

We have not performed standard histological diagnosis of CAA on the basis of the post-mortem histological findings that permit to distinct two types of CAA: CAA type 1, characterised by β-amyloid in cortical capillaries (with or without involvement of other vessels) and CAA type 2, where β-amyloid deposits are restricted to leptomeningeal and cortical arteries, arteriolesand, rarely, veins [2, 3].

## METHODS

We enrolled all consecutive patients admitted to Neurosurgery Ward of University Hospital “P. Giaccone” of Palermo with a diagnosis of spontaneous non-hypertensive ICH from December 2014 to January 2016.

The diagnosis of non-hypertensive intracerebral haemorrhage was based on these criteria [64]: -lobar anatomic location; -extra-pontine anatomic location (ganglionic, thalamic or cerebellar); -no diagnostic evidence of small vascular malformations (intracerebral arteriovenous malformations, cavernous angiomas, or venous angioma); -no diagnostic evidence of brain tumors; -no history of a previous treatment with pro-haemorragic drugs such as anticoagulants, amphetamines and other sympathomimetic drug.

Control subjects comprised 12 histologically normal temporal artery samples from 12 consecutive patients (7 women, 5 men; median 74 years, range: 60–84 years) enrolled in a previous study [65] that recruited patients with suspected giant cell arteritis but with negative biopsy results.

Control patients had no clinical history of cerebrovascular accidents (*TIA, ischemic stroke and ICH*) or Alzheimer Disease (AD).

The diagnosis in control patients was: fever of unknown origin (five patients), isolated polymyalgia rheumatica (five patients), non-specific headache in the presence of osteoarthritis (five patients).

This study has been approved by the Ethics Committee of the University of Palermo. Signed informed consent for the collection and storage of biological material has been also obtained from all the patients enrolled in this study. All patients gave their informed consent before enrolment into the study.

### Temporal biopsy

Biopsy of superficial temporal artery was performed by a specialist neurosurgeon under local anesthesia if there are no contraindications. The superficial temporal arteries were palpated bilaterally and if the vessel is not easily palpable, hand-held Doppler can be used to localize it. Once the artery is identified, the surgical site is marked. Intraoperative view of excised temporal artery. A specimen of at least 2 cm *in vivo* has been harvested.

### Histological evaluation

Biopsy specimens were evaluated by two experienced pathologists who had no access to clinical data. Artery biopsy specimens showed a range in length from 0.5 cm to 3.1 cm.

Amyloid deposits were detected by Congo Red staining. Congo Red staining can then be viewed under plane-polarized light. Amyloid plaques should give apple-green birefringence, usually as a Maltese cross.

The specimens were stained with H&E, PAS, Congo red and immunohistochemically with two antibodies: anti Aβ 817 (DAKO, 1: 50), actin (SMA, DAKO, 1: 50). Under microscopic evaluation the temporal artery specimen was considered positive for CAA when it showed at a yellowgreen birefringence under polarized light.

After histological review, biopsy specimens were classified into one of the following three categories: 1) β-amyloid presence; 2) no β-amyloid amyloid presence; 3) atherosclerotic disease.

To quantify β-amyloid we used Vonsattel method. Vonsattel *et al*. [23] graded CAA with regard to the severity of pathological changes in a given blood vessel: 1-mild, β-amyloid is restricted to the tunica media without significant destruction of smooth muscle cells; 2- moderate, the tunica media is replaced by β-amyloid and is thicker than normal; 3-severe, extensive β-amyloid deposition with focal wall fragmentation or even double barrelling of the vessel wall, microaneurysm formation, fibrinoid necrosis, and leakage of blood through the blood vessel wall.

The estimated proportion of β-amyloid deposit involvement in each blood vessel was recorded on the Mountjoy scale from 0 to 4. A score of 0 indicated the absence of β-amyloid. The finding of β-amyloid deposits of up to one-quarter of the vessel circumference was score 1. A score 2 marked β-amyloid deposits in up to one-half of the vessel circumference. The involvement of β-amyloid of up to three-quarters of the vessel circumference was score 3. Finally, score 4 indicated the total involvement of the vessel circumference [24].

### Apolipoprotein E genotyping

ApoE genotyping was performed, blind to the histological assessments, using a hot start polymerase chain reaction (PCR) method that had been optimized for archival formalin-fixed- paraffin-embedded tissue [66].
